# Organ-at-risk sparing with dynamic trajectory radiotherapy for head and neck cancer: comparison with volumetric arc therapy on a publicly available library of cases

**DOI:** 10.1186/s13014-022-02092-5

**Published:** 2022-07-15

**Authors:** Jenny Bertholet, Paul-Henry Mackeprang, Silvan Mueller, Gian Guyer, Hannes A. Loebner, Yanick Wyss, Daniel Frei, Werner Volken, Olgun Elicin, Daniel M. Aebersold, Michael K. Fix, Peter Manser

**Affiliations:** 1 Division of Medical Radiation Physics and Department of Radiation Oncology, Inselspital, Bern University Hospital and University of Bern, Friedbühlschulhaus, 3010 Bern, Switzerland; 2 Department of Physics, ETH Zurich, Zurich, Switzerland

**Keywords:** Treatment planning, Non-coplanar radiotherapy, Head and neck cancer, VMAT, OAR sparing

## Abstract

**Background:**

Dynamic trajectory radiotherapy (DTRT) extends volumetric modulated arc therapy (VMAT) with dynamic table and collimator rotation during beam-on. The aim of the study is to establish DTRT path-finding strategies, demonstrate deliverability and dosimetric accuracy and compare DTRT to state-of-the-art VMAT for common head and neck (HN) cancer cases.

**Methods:**

A publicly available library of seven HN cases was created on an anthropomorphic phantom with all relevant organs-at-risk (OARs) delineated. DTRT plans were generated with beam incidences minimizing fractional target/OAR volume overlap and compared to VMAT. Deliverability and dosimetric validation was carried out on the phantom.

**Results:**

DTRT and VMAT had similar target coverage. For three locoregionally advanced oropharyngeal carcinomas and one adenoid cystic carcinoma, mean dose to the contralateral salivary glands, pharynx and oral cavity was reduced by 2.5, 1.7 and 3.1 Gy respectively on average with DTRT compared to VMAT. For a locally recurrent nasopharyngeal carcinoma, D_0.03 cc_ to the ipsilateral optic nerve was above tolerance (54.0 Gy) for VMAT (54.8 Gy) but within tolerance for DTRT (53.3 Gy). For a laryngeal carcinoma, DTRT resulted in higher dose than VMAT to the pharynx and brachial plexus but lower dose to the upper oesophagus, thyroid gland and contralateral carotid artery. For a single vocal cord irradiation case, DTRT spared most OARs better than VMAT. All plans were delivered successfully on the phantom and dosimetric validation resulted in gamma passing rates of 93.9% and 95.8% (2%/2 mm criteria, 10% dose threshold).

**Conclusions:**

This study provides a proof of principle of DTRT for common HN cases with plans that were deliverable on a C-arm linac with high accuracy. The comparison with VMAT indicates substantial OAR sparing could be achieved.

**Supplementary Information:**

The online version contains supplementary material available at 10.1186/s13014-022-02092-5.

## Background

Radiation therapy plays an important role in the management of head and neck (HN) cancer but is often challenging, especially for target volumes with complex shapes overlapping with organs-at-risk (OARs). The introduction of intensity-modulated radiotherapy (IMRT) has enabled clinically significant toxicity reduction through better dosimetric sparing of OARs [1] while volumetric modulated arc therapy (VMAT) improved delivery efficiency with dynamic gantry rotation [2].

Non-coplanar radiotherapy can further improve OAR sparing [3] with, e.g., 4π-IMRT using up to 30 non-coplanar beams [4], non-coplanar VMAT with multiple arcs at static non-coplanar table angles [5], or non-coplanar dynamic trajectory radiotherapy (DTRT) with simultaneous gantry and table rotation during beam-on, with [6–8] or without [9, 10] dynamic collimator rotation. The trade-off between estimated delivery time and dosimetric plan quality was explored for nasopharyngeal tumours finding non-coplanar dynamic trajectories to be dosimetrically beneficial over coplanar techniques at the cost of longer, yet acceptable, delivery times [11].

Despite encouraging dosimetric quality of DTRT plans and the promise of deliverability on standard C-arm linacs [6, 12], it remains a research topic for HN radiotherapy and is not yet clinically available. Nasopharyngeal and cranial tumours have often been investigated owing to the large collision-free space [7, 11, 12] with static-table non-coplanar solutions already commercially available on C-arm linacs [13, 14]. Large HN target volumes are often associated with high rates of toxicity and could benefit from DTRT but these have a more caudal isocenter. The resulting collision-free space is more restrictive than for nasopharyngeal or cranial tumours and requires careful consideration for deliverability [15].

A practical approach to determine dynamic table paths is the use of geometric criteria to minimize target/OAR overlap by combining gantry-table cost-maps of various OARs in one map where a path-finding algorithm returns the path of lowest cost [6, 9]. However, for HN, there are many OARs that may overlap with the target, and optimal OAR selection and/or weighting at the path-finding stage remains unclear. Additionally, this provides only one path whereas multiple arcs are recommended for VMAT [16, 17]. Selecting and grouping OARs in different maps to generate more than one path for DTRT planning would enable to better exploit the collision-free space.

The aim of this proof-of-principle study was to establish path-finding strategies for DTRT of HN cases, evaluate OAR sparing compared to state-of-the-art VMAT, and demonstrate DTRT deliverability and dosimetric accuracy. For this purpose, a publicly available library covering all common HN cases was created on an anthropomorphic phantom.

## Methods

### Library of cases and clinical goals

The library of HN cancer cases was created on an axial computed tomography (CT) scan of the Alderson phantom (Radiology Support Devices Inc., USA). The phantom was immobilized in a 5-point thermoplastic mask (Posifix, civco Radiotherapy Inc., USA) and scanned on a Philips Brilliance Big Bore CT-scanner (Philips Healthcare, Amsterdam, The Netherlands) with 2 mm slices and 512 × 512 pixels in-plane resolution. All relevant OARs were delineated according to guidelines [18, 19]. Both hippocampi were additionally contoured [20].

Seven typical HN cases were identified, six of whom had elective nodal volumes treated to 50.00 Gy in 2 Gy-fractions. Sequential boost volumes are prescribed a total of 66.00 Gy for any post-operative positive margin and nodal levels with extranodular extension and to 70.00 Gy for non-operated primary tumour and involved lymph nodes [21–28].

The clinical target volumes (CTV) were delineated on the phantom based on commonly observed clinical cases and relevant guidelines [29–31]. Water density was assigned to air in the CTVs or where tumour infiltration would replace bone.

Corresponding planning target volumes (PTV) were obtained by applying a 3 mm isotropic margin around the CTV, trimmed 3 mm from body contour according to institutional practice. Plans for individual phases were normalized such that PTV_D95%_ = 100% of the prescribed dose. OAR clinical goals are summarized in Additional file 1: table A.II [32]. The hippocampus constraint was set at D_40%_ < 7.3 Gy [33]. All OARs were the same for these six cases.

The seventh case was an early stage glottic laryngeal carcinoma treated with single vocal cord irradiation (SVCI) to 58.08 Gy in 16 fractions [34]. OAR delineation and planning protocol are described elsewhere (VoiceS NCT04057209) and summarized in Additional file 1: table A.IV.

### DTRT paths and VMAT arcs set-up

All treatment plans were created for 6 MV-flattened beam on a TrueBeam linac (Varian Medical Systems) equipped with a 120-leaf Millennium multi-leaf collimator (MLC) and a PerfectPitch 6-degree-of-freedom table.

DTRT plans were created based on Fix et al. [6]. In short, target and OAR contours are exported from Eclipse (Varian, research version 15.6) to an in-house path-finding software using the research Eclipse Scripting Application Programming Interface (ESAPI). For each OAR, a gantry-table (GT) cost-map is generated quantifying the fractional target/OAR volume-overlap in beam's eye view for each combination of gantry-table angle, accounting for the relative position of the OAR with respect to the target. OAR maps are combined in a weighted sum and exclusion zones are determined based on collision and CT-scan length restrictions.

An A* path-finding algorithm is used to determine the GT-path of lowest cost for a given range of gantry rotation. For the chosen GT-path, a collimator-gantry (GC) map is created that quantifies field width in the x-direction. The A* is used to the determine the GC-path of lowest cost, thereby reducing the range of possible leaf-travel. The selected gantry-table-collimator (GTC)-paths are imported back into Eclipse via ESAPI for intensity modulation optimization.

In this study, case-specific collision maps were determined based on a validated virtual linac model using Blender [35, 36]. The model detects possible collisions between the gantry and the table-top/table-stand and a patient model. A reference point on the headrest and the plan isocenter coordinates are used to estimate the table position in the room. An additional safety margin of 2 cm on each component was used.

All DTRT paths covered a full gantry rotation with control points every 2°. The A* algorithm was restricted to find GT and GC-paths with a maximum gradient of 3° table/collimator rotation per degree gantry rotation and paths were smoothed using a 10-points (20°) moving average to avoid abrupt table motion. Different paths per plan were created by selecting different OARs to generate each map. Individual OAR-maps were equally weighted. To increase the degrees-of-freedom at the intensity optimization stage, some paths were duplicated either by field-splitting in the x-direction using the secondary collimator jaws or by applying a constant 90° collimator offset to the A*-determined GC-path.

For each DTRT plan, a VMAT plan was created with the same number of full arcs as GT-paths, the same field-splitting strategy, and collimator angle of 5 or 95°.

### Intensity optimization and dose calculation

Research version of the Eclipse photon optimizer (PO) and the Anisotropic Analytical Algorithm (AAA) version 15.6 were used for intensity modulation optimization and dose calculation with a 2.5 mm grid. Intermediate dose calculation was used, "convergence mode" was on, and "aperture shape controller" set to moderate [37].

A set of manual planning rules was designed to minimize planner bias (Additional file 1: A.I), where the optimization objectives are found during interactive planning, but only certain parameters can be changed. Each plan was optimized once and then re-optimized without any manual interaction. Objective tweaking and re-optimization without manual interaction was allowed if clinical goals were nearly reached. After final dose calculation with AAA, plans were normalized. All plans were optimized by the same planner.

### Treatment technique comparison

Plans were reviewed for clinical acceptability by a radiation oncologist. PTV coverage, homogeneity index (HI_95%_ = V_95%_-V_105%_), and Paddick conformity index (CI_Paddick_ [38]) for each individual dose level were evaluated, but technique comparison focused on OAR dose in the dose distributions for the combined plans.

### Deliverability of DTRT plans

To demonstrate deliverability of DTRT, all plans were delivered in developer mode using xml files and machine log-files were recorded to evaluate mechanical accuracy and the possible correlation between speed and mechanical deviations for each dynamic axis. Prior to delivery, the phantom was positioned on the TrueBeam PerfectPitch table with the thermoplastic mask. Orthogonal kV imaging was used to adjust patient positioning with 5 degrees-of-freedom. Rotation was not corrected because the dynamic table rotation is encoded for each control point in the xml files and any correction would be overridden during delivery.

Dosimetric validation with film measurement was performed for one case as described in Additional file 1: A.II [39–42].

Recently, the Radiotherapy Treatment plannINg study Guidelines (RATING) were proposed to evaluate the quality of planning studies and there reporting. The guidelines were followed and the RATING score was calculated [43].

## Results

The seven HN cases are presented in Table 1. The library consisting of the Alderson CT-scan and structure-sets for each case is publicly available in DICOM format on the BORIS repository (https://dx.doi.org/10.48350/159243).

**Table 1:** Library of case, DTRT paths and VMAT arc set up, and delivery time

Case/dose level	PTV volume (cc)	DTRT paths and OAR selection	VMAT arcs	Delivery time (min)		
				DTRT		VMAT
**HN1**	Locoregionally advanced oropharyngeal carcinoma (bilateral elective nodal irradiation)					
50.00 Gy	592.6	2 × hippo. R + L, oral cavity, parotid R + L, brain stem PRV (SF)	2 × C. 5° (SF)	10.4		4.0
		2 × hippo. R + L, pharynx, contr. submand., spinal cord PRV (SF)	2 × C. 95° (SF)			
70 Gy	64.0	Same as 50 Gy level		9.2		4.0
**HN2**	Locoregionally advanced oropharyngeal carcinoma (bilateral elective nodal irradiation)					
50.00 Gy	560.8	2 × hippo. R + L, oral cavity, parotid R + L, brain stem PRV (SF) (see Fig. 1, top left)	2 × C. 5° (SF)	9.6		4.0
		2 × hippo. R + L, pharynx, contr. submand., spinal cord PRV (SF) (see Fig. 1, bottom left)	2 × C. 95° (SF)			
66.00 Gy	189.5	2 × hippo. R + L, oral cavity, parotid R + L, brain stem PRV (SF)	2 × C. 5° (SF)	6.9		3.0
		1 × hippo. R + L, pharynx, contr. submand., spinal cord PRV	1 × C. 95°			
70.0 Gy	134.4	2 × hippo. R + L, pharynx, oral cavity, parotid R + L, contr. submand., spinal cord PRV, brain stem PRV (CRot)	1 × C. 5° 1 × C. 95°	5.0		2.0
**HN3**	Locoregionally advanced oropharyngeal carcinoma (unilateral elective nodal irradiation)					
50.00 Gy	279.3	2 × hippo. R + L, pharynx, oral cavity, parotid R + L, submand. R + L, spinal cord PRV, brain stem PRV (SF)	2 × C. 5° (SF)	7.5		3.0
		1 × hippo. R + L, pharynx, contr. carotid PRV	1 × C. 95°			
70.0 Gy	47.3	1 × hippo. R + L, pharynx, oral cavity, parotid R + L, submand. R + L, spinal cord PRV, brain stem PRV (SF)	1 × C. 5°	5.0		2.0
		1 × hippo. R + L, pharynx, contr. carotid PRV	1 × C. 95°			
**HN4**	Adenoid cystic carcinoma of the left parotid gland (postoperative) (ACC)					
50.0 Gy	96.1	2 × hippo. R + L, oral cavity, pharynx, contr. parotid, submand. R + L, spinal cord PRV, brain stem PRV (CRot)	1 × C. 5° 1 × C. 95°	5.6		2.0
66.00 Gy	32.8	Same as 50 Gy level		4.5		2.0
**HN5**	Locally recurrent nasopharyngeal carcinoma					
50.00 Gy	66.7	1 × hippo. R + L, eye R + L	1 × C. 5°	6.5		3.0
		1 × hippo. R + L, optic nerve R + L, chiasm, lens R + L	1 × C. 5°			
		1 × hippo. R + L, carotid PRV R + L, lens R + L	1 × C. 95°			
66.00 Gy	55.7	Same as 50 Gy level		7.1		3.0
**HN6**	Stage II laryngeal carcinoma (no elective neck volume)					
50.00 Gy	39.1	2 × normal tissue*, pharynx, contr. carotid PRV, upper oesophagus, thyroid (CRot)	1 × C. 5° 1 × C. 95°	4.9		2.0
70.00 Gy	20.7	Same as 50 Gy dose level		4.9		2.0
**HN7**	Early stage glottic laryngeal carcinoma (single vocal cord irradiation, SVCI)					
58.08 Gy	9.7	2 × normal tissue*, inferior constrictor (CRot)	1 × C. 5° 1 × C. 95°	4.9		2.3

^*^Normal tissue is the body volume excluding PTV

*R* right, *L* left, *hippo*. hippocampus, *submand*. submandibular gland, *C* collimator, *SF* split field, *CRot* collimator rotation, i.e. offset of 90° between the 2 paths

**Fig. 1:**
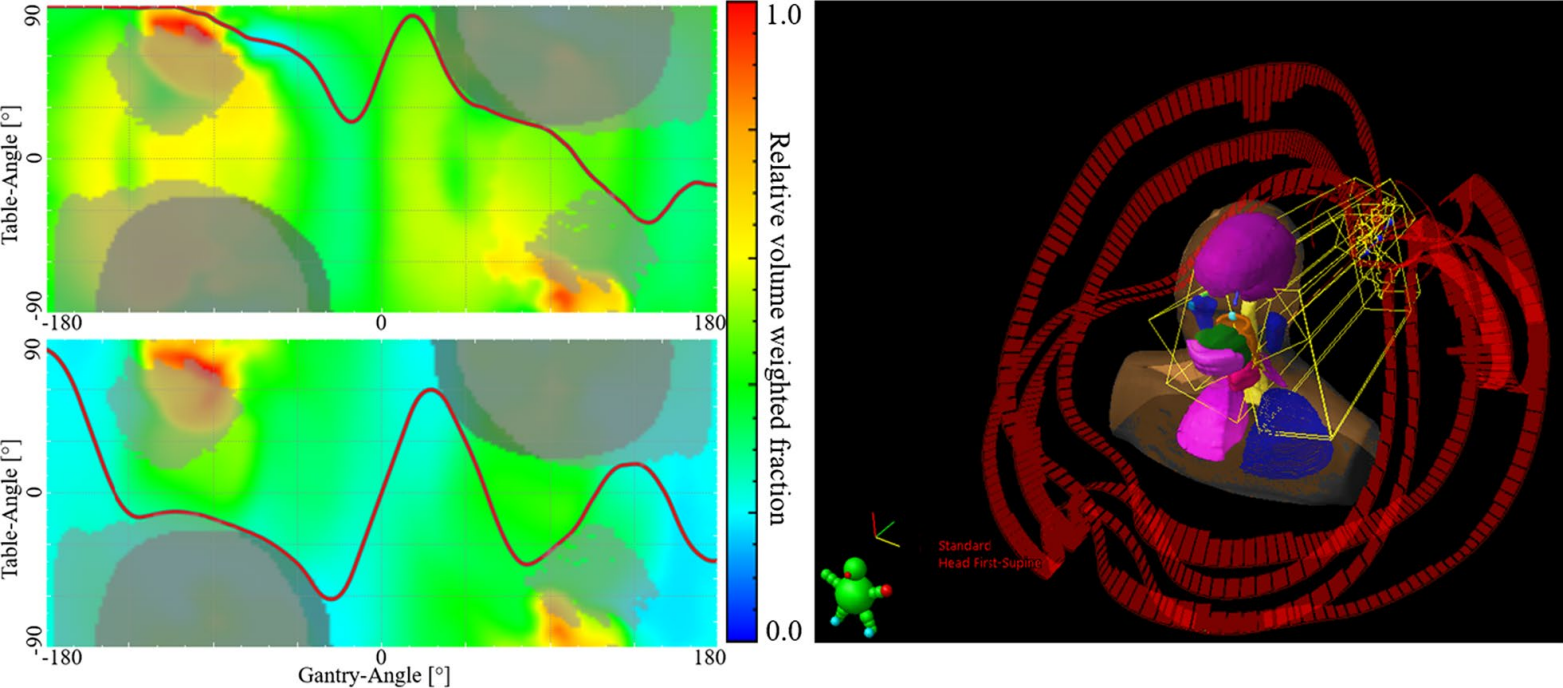
Example GT-maps with A*-determined paths (red curve) in the path-finder framework (left) and imported into Eclipse for the 50 Gy dose level of HN2. Light grey areas indicate collision zones, dark grey areas indicate end-of-CT restrictions. OAR selection for each map is detailed in Table 1. Individual OAR maps are shown in Additional file 1: Fig. 1

OAR selection strategies for each case were determined empirically to obtain DTRT paths covering the 4π-space while avoiding the most relevant OARs (Table 1). Example GT-maps and paths are shown in Fig. 1 with the corresponding individual OAR GT-maps in Additional file 1: Fig. 1. The process from contour export to the in-house software to paths import in Eclipse currently takes approximately 8–12 min for a 2–4 paths plan.

Each individual plan and dose distributions from all combined plans were considered clinically acceptable by a radiation oncologist. Target coverage were similar between DTRT and VMAT. For conventional fractionation (HN1-6) CI_Paddick_ were between 0.82 and 0.92 differing at most by 0.03 between DTRT and VMAT. HI_95%_ were between 74.5% and 99.2% with a mean absolute difference of 3.2% between DTRT and VMAT (VMAT being generally more homogeneous).

Target coverage in the dose distributions for the combined plans were similar for the high dose volume but differences were observed for lower dose volumes depending on the direction in which the elective volume extended the high dose volume (Additional file 1: Fig. 2).

**Fig. 2:**
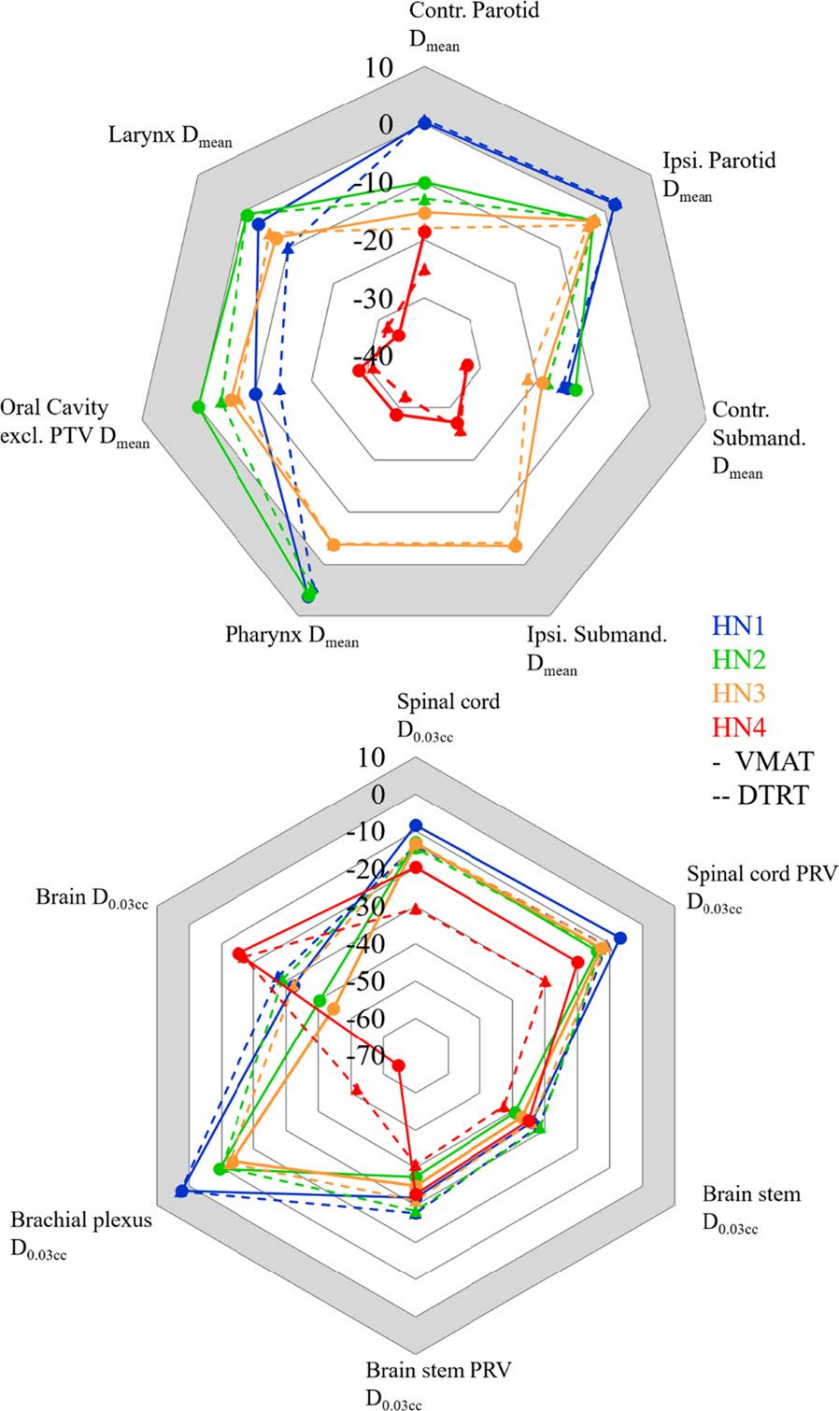
Radar plots showing the difference in OAR dose compared to tolerance for HN 1–4 for VMAT (solid lines) and DTRT (dashed lines). Mean doses are considered for the salivary glands, pharynx, oral cavity and larynx (top) and near max doses are considered for the nervous system (bottom). Negative values indicate better sparing than tolerance. Positive values (grey shaded area) indicate doses above tolerance. *Ipsi*: ipsilateral, *Contr*. contralateral, *Submand*: Submandibular (gland), *excl*: excluding

Dosimetric endpoints of the dose distributions for the combined plans are reported in Additional file 1: table A.III and IV. All plans had acceptable target coverage and near-max dose. Mandatory clinical goals were fulfilled without compromising target coverage. For some OARs, the dose was above tolerance but within acceptable deviations.

**Fig. 3:**
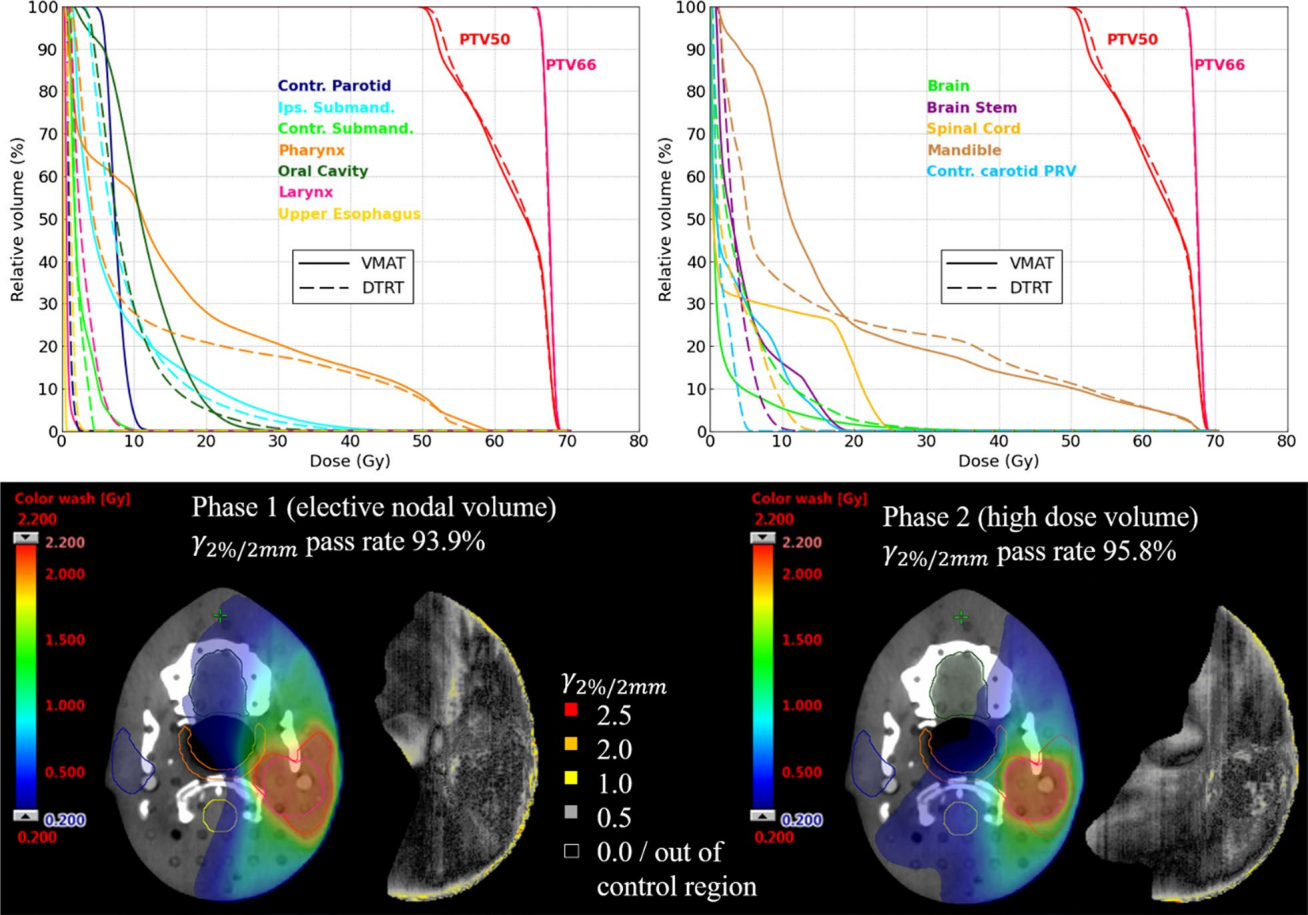
DVHs for HN4 (top) for VMAT (solid line) and DTRT (dashed line). Dose distribution from Eclipse and corresponding gamma maps (2%/2 mm, threshold: 10% of maximum dose) comparing film dose to AAA-calculated dose in Eclipse for DTRT for both dose levels (bottom)

For the oropharyngeal cases and the ACC (HN1-4), challenging OARs were the salivary and swallowing structures. Mean dose to the contralateral salivary glands was on average 2.5 Gy lower for DTRT than for VMAT; it was on average 1.7 Gy and 3.1 Gy lower for the pharynx and oral cavity respectively (Fig. 2). Dose to the auditory or optic structures was generally higher for DTRT than VMAT (Additional file 1: table A.IV) but at least 22 Gy below tolerance except for the lenses where it was at least 0.4 Gy below the tolerance of D_0.03 cc_ ≤ 6 Gy. V_7.3 Gy_ to the hippocampi was higher for DTRT than for VMAT but remained well below tolerance (maximum 28.5%). Dose volume histograms (DVHs) for HN4 are shown in Fig. 3 (top).

**Fig. 4:**
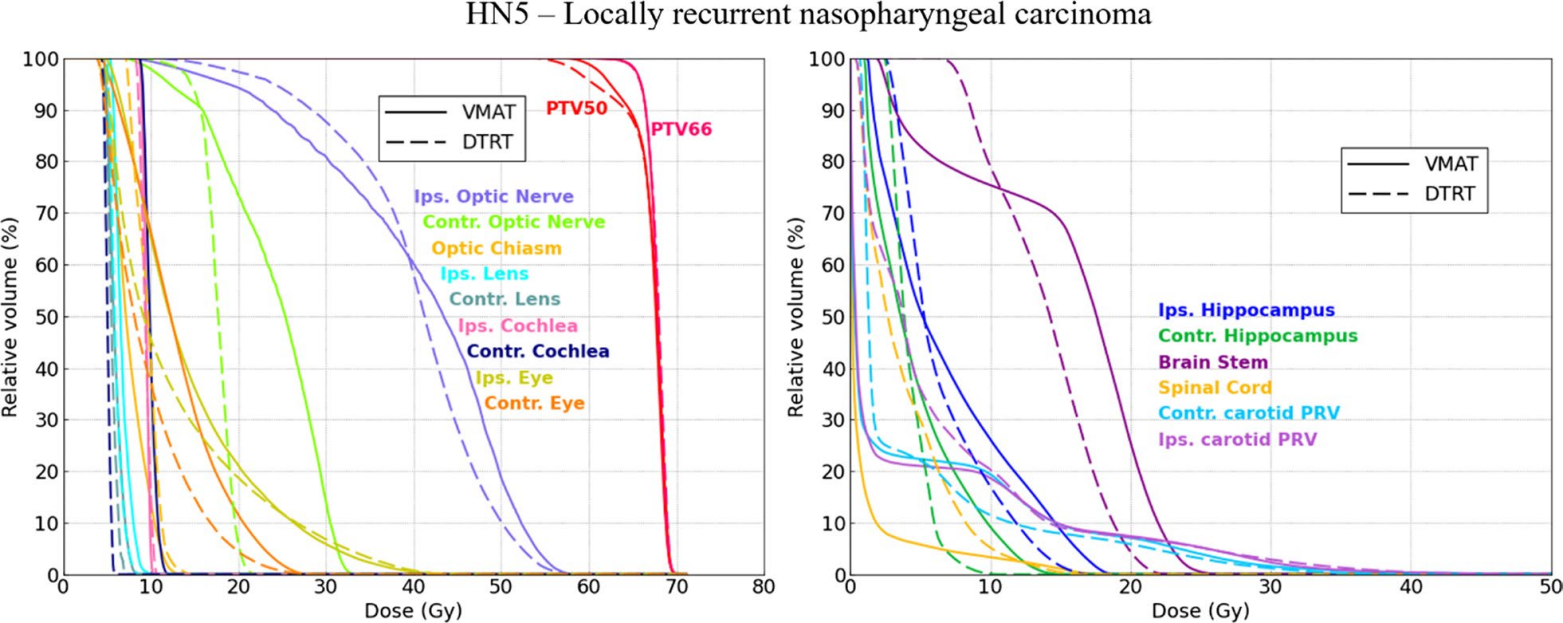
DVHs plots for HN5, locally recurrent nasopharyngeal carcinoma

For the nasopharyngeal case (HN5), challenging OARs were the optic and visual structures. DVHs are shown in Fig. 4. Near maximum dose to the ipsilateral optic nerve was above tolerance (54.0 Gy) for VMAT (54.8 Gy) but within tolerance for DTRT (53.3 Gy). Better OAR sparing for DTRT compared to VMAT was achieved for most endpoints (Additional file 1: table A.III).

**Fig. 5:**
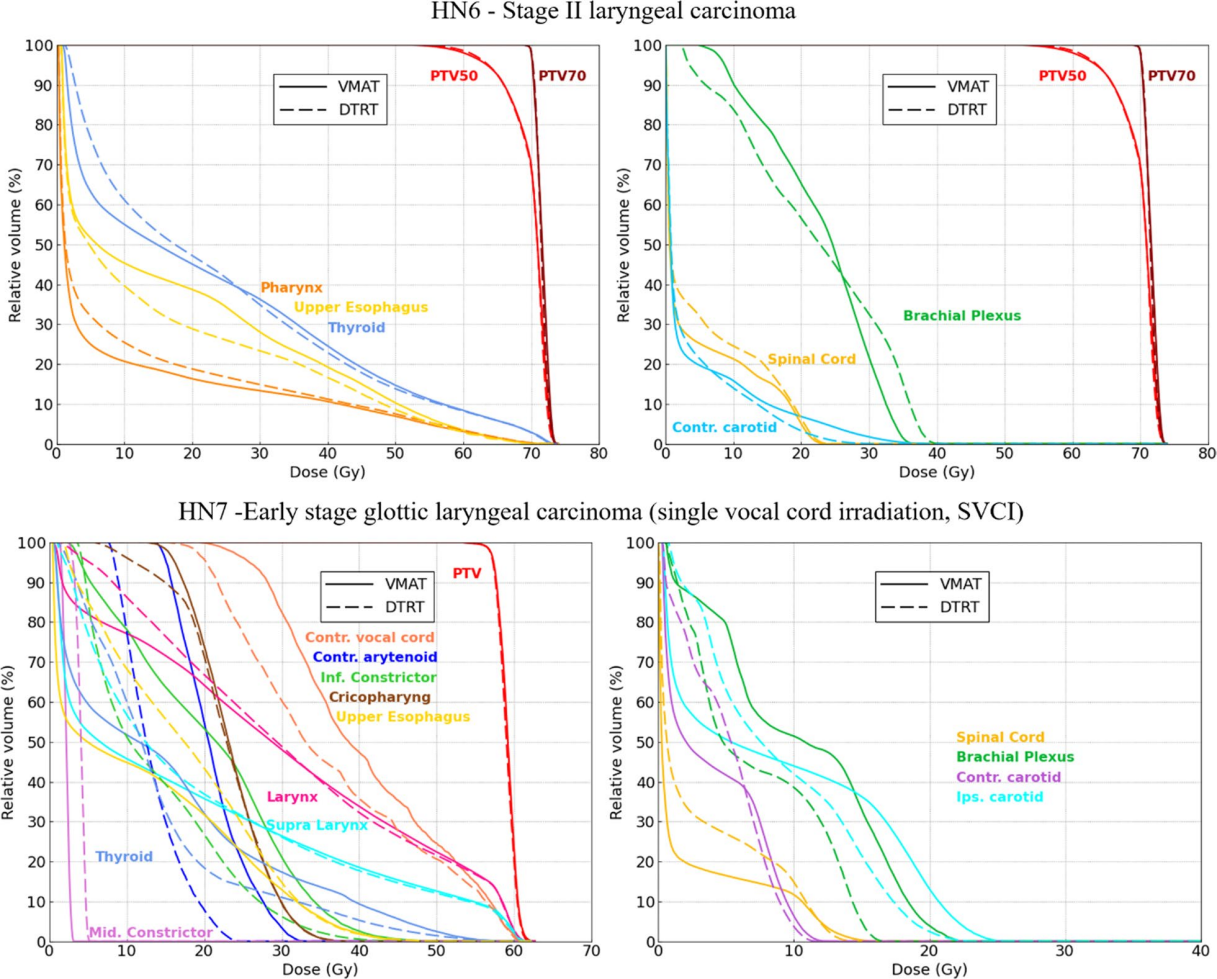
DVH plots for the laryngeal cases HN6 and HN7 (SVCI). Parallel OARs are shown on the left, serials OARs are shown on the right

DVHs for the laryngeal cases (HN6-7) are shown in Fig. 5. For HN6, mean dose to the pharynx was 10.4 Gy (DTRT) and 9.3 Gy (VMAT); it was 15.5 Gy (DTRT) and 17.9 Gy (VMAT) for upper oesophagus. D_50%_ to the contralateral carotid PRV was 14.0 Gy (DTRT) and 15.0 Gy (VMAT). For the SVCI case, HN7, the plan was normalized such that PTV_D97%_ = 99% to fulfil the prescription for both plans. CI_Paddick_ and HI_95%_ were 0.71 and 96.0% for DTRT and 0.77 and 96.8% for VMAT. DTRT achieved better OAR sparing than VMAT for most OARs (Additional file 1: table A.IV).

All plans were successfully delivered on the Alderson phantom in developer mode (Additional file 3: video). Delivery times, calculated from the machine log-files, were on average 2.4 (range: 2.1–2.8) times longer for DTRT compared to VMAT (Table1). The mechanical accuracy for each moving component is reported as the difference between expected and actual value in the machine log-files in Table 2. There was a high correlation (Pearson’s correlation coefficient) between speed and deviations for table and collimator angles, indicating that the table and collimator tend to lag behind their respective expected position, however correlation between speed and deviations was low for gantry angle.

**Table 2:** Deviations between expected and actual angle/position for DTRT deliveries

Axis	Root-mean-square (RMS) difference	Maximum difference	Correlation between speed and deviation
Gantry angle (°)	0.02	0.13	0.16, *p* < < 0.01
Table angle (°)	0.12	0.16	< -0.99, *p* < < 0.01
Collimator angle (°)	0.03	0.17	< -0.99, *p* < < 0.01
	*RMS deviation over all moving leaves*		
	Mean RMS	Max RMS	
MLC leaves position (mm)	0.17	0.28	

Figure 3 shows the results of the dosimetric validation for HN4. The gamma passing rates (global 2%/2 mm, 10% dose threshold) were 93.9% and 95.8% with failing pixels located mostly at the film border.

The overall RATING score was 96%. RATING scores were verified during review. (Additional file 2: A.V).

## Discussion

This proof-of-principle study indicates substantially improved OAR sparing with equivalent target coverage may be achieved for HN radiotherapy with DTRT compared to VMAT. For three oropharyngeal carcinomas (HN1-3) and one adenoid cystic carcinoma (HN4), the reduction in mean dose to the contralateral salivary glands (2.5 Gy, average), pharynx (1.7 Gy), and oral cavity (3.1 Gy) has the potential to reduce xerostomia and dysphagia, with a positive impact on quality of life [44].

Few studies had previously investigated non-coplanar radiotherapy for HN cases with bilateral elective nodal irradiation volumes. Krayenbuehl et al. [10] reported improvement in OAR sparing of 2.9 Gy on average for parotid glands, 2.4 Gy for the oral mucosa and 6.9 Gy for the larynx using non-coplanar arcs compared to 5-beam IMRT in ten patients. Gayen et al. [45] have found non-coplanar VMAT to be advantageous over coplanar VMAT both in sequential boost and simultaneous integrated boost techniques for sparing the shoulders and improving target coverage in 22 patients. Subramanian et al. [5] compared coplanar VMAT to multi-isocentric non-coplanar VMAT in 25 patients obtaining average reductions in mean dose to the parotids, larynx, oral cavity and pharyngeal muscle between 3 and 5 Gy. This improved sparing may be partly because non-coplanar plans had 1.5–3 times as many arcs as coplanar ones whereas, in the present study, DTRT and VMAT plans had the same number of arcs/paths.

For a nasopharyngeal case (HN5), DTRT resulted in lower dose to the optic structures compared to VMAT. In particular, near-max dose to the ipsilateral optic nerve was above tolerance for VMAT but within tolerance for DTRT, where the dose-volume effect for radiation-induced optic neuropathy risk is rapidly increasing [46]. Near-max dose to the lenses was slightly reduced with DTRT but above tolerance for both plans. The volume of the ipsilateral hippocampus receiving more than 7.3 Gy was 38.0% for VMAT and 30.5% for DTRT. Tolerance would have likely been exceeded for VMAT in a clinical setting where this OAR is generally not considered with an associated risk of neurocognitive impairment [33]. The hippocampus should be considered in the planning of nasopharyngeal cases with both coplanar and non-coplanar techniques.

The dosimetric benefit of DTRT for stage II laryngeal carcinoma (HN6) was unclear with DTRT resulting in higher doses than VMAT for the pharynx and brachial plexus but improved sparing for upper oesophagus, thyroid gland, and contralateral carotid artery. A recent analysis found no difference in survival for IMRT or 3D-conformal radiotherapy for early-stage laryngeal carcinoma but toxicity was not reported [47]. For the more advanced SVCI technique in early stage glottic cancer (HN7), DTRT resulted in improved sparing for most OARs compared to VMAT. Historically, transoral surgery and standard radiotherapy have been associated with comparable morbidity [48, 49] indicating that improved OAR sparing with DTRT and SVCI could surpass surgery.

One possible limitation of this study is planner bias due to manual planning [43]. All VMAT plans were created for the purpose of this study using the same number of arcs as DTRT paths and comparable field-splitting and collimator angle offset strategies. To further mitigate planner bias during intensity optimization, strict manual planning rules were set before planning commenced (Additional file 1: A.I) and all plans were created by the same planner. Although using the same optimization objectives for both plans could be perceived as bias mitigation and would enable to compare the objective function value [11], this would not allow to explore the true potential for OAR sparing of one technique over the other. Automated planning is an attractive approach to mitigate bias [50] but no suitable method is currently available for DTRT on our system.

To exploit the OAR sparing potential of DTRT, OAR grouping strategies to obtain different non-coplanar paths were developed through trial-and-error. This approach is a priori applicable to other geometry-based path-finding approaches [7, 9] but intensity modulation is not considered at the path-finding stage. Other approaches use 4π fluence-based optimization to inform path-finding [11, 51, 52] or simultaneous path and intensity modulation optimization [53, 54] which may further improve dosimetric plan quality. However, the two-step geometry-based DTRT treatment planning process is less complex and compatible with Eclipse making it potentially easier to implement clinically.

All DTRT paths were created using case-specific collision models automatically generated on a virtual linac and patient model [35] to optimally yet safely exploit the 4π-space around the patient. All plans were deliverable on the anthropomorphic phantom. The model could be refined using patient-specific body contours and mensuration or surface scanning [15].

Deliverability and dosimetric accuracy of dynamic trajectory delivery was previously demonstrated in cubic or cylindrical phantoms [6, 12, 55] and on an anthropomorphic prostate phantom [55] while Mueller et al. demonstrated deliverability of dynamic mixed beam radiotherapy (DYMBER), combining DTRT with electron fields, on a head phantom [56]. Here, deliverability of DTRT for HN was demonstrated with the full Alderson phantom on the table. Delivery times were on average 2.4 times longer for DTRT than VMAT for the same number of full gantry rotations but remain clinically acceptable. Mechanical accuracy of the delivery was assessed as the deviations between expected and actual values in machine log-files for all mechanical components, with root-mean-square (RMS) deviation of 0.02°, 0.12° and 0.03° for the gantry, table and collimator angles respectively. Film measurements resulted in gamma passing rates of 93.9% and 95.8% (2%/2 mm criteria, 10% dose threshold) confirming that DTRT is deliverable with high mechanical and dosimetric accuracy and clinically acceptable delivery times.

There were several motivations to conduct this study on a phantom. First, patient CTs do not always extend to the vertex restricting possible beam incidences and preventing dose reporting in cut regions. Second, deliverability and dosimetric validation could be performed directly on the phantom, therefore enabling comprehensive end-to-end testing. Finally, the anthropomorphic phantom solution allowed to create a publicly available library of all common HN cases. The CT and contours can be directly used for in silico planning studies or users can register the contours to their own Alderson phantom (available in many clinics) for measurements. Given the anthropomorphism of the phantom, the proposed DTRT planning strategy is expected to be applicable to real patients presenting with similar target shapes and location to these available in the library. On the other hand, it may be possible to favour sparing specific OARs on a case-by-case basis.

## Conclusions

This study showed substantial improvement in OAR sparing for HN cancer radiotherapy using DTRT compared to VMAT with plans that are deliverable on standard linacs. Film measurements for one case showed good agreement with the calculated dose. A publicly available library of all common HN cancer cases was created and the treatment planning strategy applied on these cases can be applied to similar cases in future clinical studies, therefore bringing DTRT closer to clinical practice.

## Supplementary Information

**Additional file 1: A.I**. Manual planning rules; **Table A.I** Optimization objectives for target coverage and conformity; **Table A.II**. Clinical goals and starting optimization objectives for OARs. **A.II**. Film measurement protocol; **A.III** DVH endpoints; **Table A.III** DVH endpoints for HN1-6; **Table A.IV** DVH endpoints for HN7.

**Additional file 2: A.V**. RATiNG.

**Additional file 3: A.VI**. Video of DTRT delivery.

## Data Availability

The datasets generated and analysed during the current study are available on the BORIS repository. https://dx.doi.org/10.48350/159243

## Abbreviations

AAA: Analytical anisotropic algorithm
ACC: Adenoid cystic carcinoma
CI: Conformity index
CT: Computed tomography
CTV: Clinical target volume
DTRT: Dynamic trajectory radiotherapy
DVH: Dose volume histogram
DYMBER: Dynamic mixed beam radiotherapy
ESAPI: Eclipse scripting application programming interface
GT: Gantry-table
GC: Gantry-collimator
GTC: Gantry-table-collimator
HI: Homogeneity index
HN: Head and neck
IMRT: Intensity modulated radiation therapy
kV: Kilo-voltage
MLC: Multi-leaf collimator
NPC: Nasopharyngeal carcinoma
OAR: Organ at risk
PO: Photon optimizer
RATING: Radiotherapy treatment planning study guidelines
SVCI: Single vocal cord irradiation
VMAT: Volumetric modulated arc therapy

## References

[1] Nutting CM, Morden JP, Harrington KJ, Urbano TG, Bhide SA, Clark C, et al. Parotid-sparing intensity modulated versus conventional radiotherapy in head and neck cancer (PARSPORT): a phase 3 multicentre randomised controlled trial. 2011;127–36. 10.1016/S1470-2045(10)70290-4 PMC3033533 21236730

[2] Otto K (2008). Volumetric modulated arc therapy: IMRT in a single gantry arc. Med Phys.

[3] Smyth G, Evans PM, Bamber JC, Bedford JL (2019). Recent developments in non-coplanar radiotherapy. Br J Radiol.

[4] Rwigema JM, Nguyen D, Heron DE, Chen AM, Lee P, Wang P (2015). 4 pi Noncoplanar stereotactic body radiation therapy for head-and-neck cancer : potential to improve tumor control and late toxicity. Radiat Oncol Biol.

[5] Subramanian VS, Subramani V, Chilukuri S, Kathirvel M, Arun G, Swamy ST (2017). Multi-isocentric 4π volumetric-modulated arc therapy approach for head and neck cancer. J Appl Clin Med Phys.

[6] Fix MK, Frei D, Volken W, Terribilini D, Mueller S, Elicin O (2018). Part 1: optimization and evaluation of dynamic trajectory radiotherapy. Med Phys.

[7] Yang Y, Zhang P, Happersett L, Xiong J, Yang J, Chan M (2011). Choreographing couch and collimator in volumetric modulated arc therapy. Int J Radiat Oncol Biol Phys.

[8] Locke CB, Bush KK. Trajectory optimization in radiotherapy using sectioning (TORUS): Medical Physics. 2017;44:3375–92. 10.1002/mp.12270 28397968

[9] Smyth G, Bamber JC, Evans PM, Bedford JL (2013). Trajectory optimization for dynamic couch rotation during volumetric modulated arc radiotherapy. Phys Med Biol.

[10] Krayenbuehl J, Davis JB, Ciernik IF (2006). Dynamic intensity-modulated non-coplanar arc radiotherapy (INCA) for head and neck cancer. Radiother Oncol.

[11] Wild E, Bangert M, Nill S, Oelfke U (2015). Noncoplanar VMAT for nasopharyngeal tumors: plan quality versus treatment time. Med Phys.

[12] Smyth G, Evans PM, Bamber JC, Mandeville HC, Moore AR, Welsh LC, et al. Dosimetric accuracy of dynamic couch rotation during volumetric modulated arc therapy (DCR- VMAT) for primary brain tumours Dosimetric accuracy of dynamic couch rotation during volumetric modulated arc therapy (DCR-VMAT) for primary brain tumours. Physics in Medicine and Biology. IOP Publishing; 2019;64. 10.1088/1361-6560/ab0a8e PMC6877349 30808011

[13] Kadoya N, Abe Y, Kajikawa T, Ito K, Yamamoto T, Umezawa R (2019). Automated noncoplanar treatment planning strategy in stereotactic radiosurgery of multiple cranial metastases: HyperArc and CyberKnife dose distributions. Med Dosim.

[14] Ho HW, Lee SP, Lin HM, Chen HY, Huang CC, Wang SC (2020). Dosimetric comparison between RapidArc and HyperArc techniques in salvage stereotactic body radiation therapy for recurrent nasopharyngeal carcinoma. Radiat Oncol Radiat Oncol.

[15] Yu VY, Tran A, Nguyen D, Cao M, Ruan D, Low DA (2015). The development and verification of a highly accurate collision prediction model for automated noncoplanar plan delivery. Med Phys.

[16] Guckenberger M, Richter A, Krieger T, Wilbert J, Baier K, Flentje M (2009). Is a single arc sufficient in volumetric-modulated arc therapy ( VMAT ) for complex-shaped target volumes?. Radiother Oncol.

[17] Tol JP, Dahele M, Slotman BJ, Verbakel WFAR, Tol JP, Dahele M (2015). Increasing the number of arcs improves head and neck volumetric modulated arc therapy plans. Acta Oncol.

[18] Brouwer CL, Steenbakkers RJHM, Langendijk JA, Sijtsema NM (2015). Identifying patients who may benefit from adaptive radiotherapy: Does the literature on anatomic and dosimetric changes in head and neck organs at risk during radiotherapy provide information to help?. Radiother Oncol.

[19] Sun Y, Yu X-L, Luo W, Lee AWM, Wee JTS, Lee N (2014). Recommendation for a contouring method and atlas of organs at risk in nasopharyngeal carcinoma patients receiving intensity-modulated radiotherapy. Radiother Oncol.

[20] Scoccianti S, Detti B, Gadda D, Greto D, Furfaro I, Meacci F (2015). Organs at risk in the brain and their dose-constraints in adults and in children: A radiation oncologist’s guide for delineation in everyday practice. Radiother Oncol.

[21] Caudell JJ, Ward MC, Riaz N, Zakem SJ, Awan MJ, Dunlap NE, et al. Volume, dose, and fractionation considerations for IMRT-based reirradiation in head and neck cancer: a multi-institution analysis. Int J Radiat Oncol*Biol*Phys 2018;100:606–17. 10.1016/j.ijrobp.2017.11.036 PMC7269162 29413274

[22] Liu Y-P, Wen Y-H, Tang J, Wei Y, You R, Zhu X-L (2021). Endoscopic surgery compared with intensity-modulated radiotherapy in resectable locally recurrent nasopharyngeal carcinoma: a multicentre, open-label, randomised, controlled, phase 3 trial. Lancet Oncol.

[23] Mendenhall WM, Werning JW, Hinerman RW, Amdur RJ, Villaret DB (2004). Management of T1–T2 glottic carcinomas. Cancer.

[24] Deschuymer S, Nevens D, Duprez F, Daisne J-F, Dok R, Laenen A (2020). Randomized clinical trial on reduction of radiotherapy dose to the elective neck in head and neck squamous cell carcinoma; update of the long-term tumor outcome. Radiother Oncol.

[25] Rosenthal DI, Mohamed ASR, Garden AS, Morrison WH, El-Naggar AK, Kamal M, et al. Final report of a prospective randomized trial to evaluate the dose-response relationship for postoperative radiation therapy and pathologic risk groups in patients with head and neck cancer. Int J Radiat Oncol Biol Phys 2017;98:1002–11. 10.1016/j.ijrobp.2017.02.218 PMC5518636 28721881

[26] Bourhis J, Sire C, Graff P, Grégoire V, Maingon P, Calais G (2012). Concomitant chemoradiotherapy versus acceleration of radiotherapy with or without concomitant chemotherapy in locally advanced head and neck carcinoma (GORTEC 99–02): an open-label phase 3 randomised trial. Lancet Oncol.

[27] Lacas B, Carmel A, Landais C, Wong SJ, Licitra L, Tobias JS (2021). Meta-analysis of chemotherapy in head and neck cancer (MACH-NC): An update on 107 randomized trials and 19,805 patients, on behalf of MACH-NC Group. Radiother Oncol.

[28] Terhaard CHJ, Lubsen H, Rasch CRN, Levendag PC, Kaanders HHÀM, Tjho-Heslinga RE, et al. The role of radiotherapy in the treatment of malignant salivary gland tumors. Int J Radiat Oncol Biol Phys 2005;61:103–11. 10.1016/j.ijrobp.2004.03.018 15629600

[29] Grégoire V, Evans M, Le Q-T, Bourhis J, Budach V, Chen A (2018). Delineation of the primary tumour Clinical Target Volumes (CTV-P) in laryngeal, hypopharyngeal, oropharyngeal and oral cavity squamous cell carcinoma: AIRO, CACA, DAHANCA, EORTC, GEORCC, GORTEC, HKNPCSG, HNCIG, IAG-KHT, LPRHHT, NCIC CTG, NCRI NRG Oncolog. Radiother Oncol.

[30] Lee AW, Ng WT, Pan JJ, Poh SS, Ahn YC, AlHussain H (2018). International guideline for the delineation of the clinical target volumes (CTV) for nasopharyngeal carcinoma. Radiother Oncol.

[31] Biau J, Lapeyre M, Troussier I, Budach W, Giralt J, Grau C (2019). Selection of lymph node target volumes for definitive head and neck radiation therapy: a 2019 Update. Radiother Oncol.

[32] Lee AW, Ng WT, Pan JJ, Chiang C-L, Poh SS, Choi HC, et al. International guideline on dose prioritization and acceptance criteria in radiation therapy planning for nasopharyngeal carcinoma. Int J Radiat Oncol Biol Phys 2019;105:567–80. 10.1016/j.ijrobp.2019.06.2540 31276776

[33] Gondi V, Hermann BP, Mehta MP, Tomé WA. Hippocampal dosimetry predicts neurocognitive function impairment after fractionated stereotactic radiotherapy for benign or low-grade adult brain tumors. Int J Radiat Oncol Biol Phys. 2013;85:348–54. 10.1016/j.ijrobp.2012.11.031 23312272

[34] Al-Mamgani A, Kwa SLS, Tans L, Moring M, Fransen D, Mehilal R, et al. Single vocal cord irradiation: image guided intensity modulated hypofractionated radiation therapy for T1a glottic cancer: early clinical results. Int J Radiat Oncol Biol Phys 2015;93:337–43. 10.1016/j.ijrobp.2015.06.016 26264629

[35] Guyer G, Wyss Y, Bertholet J, Mackrepang, Paul-Henry Loebner H, Fix MK, Manser P, et al. Development of a collision prediction tool between gantry and table using blender. In: 63rd AAPM annual meeting [Internet]. 2021. https://w4.aapm.org/meetings/2021AM/programInfo/programAbs.php?sid=9375&aid=58124

[36] Blender Online Community blender. org. Blender—a 3D modelling and rendering package blender.org [Internet]. Stitching Blender Foundation, Amsterdam; 2018 [cited 2021 Oct 26]. Available from: blender.org

[37] Rossi M, Boman E. The use of aperture shape controller and convergence mode in radiotherapy treatment planning. J Radiother Pract 2020;1–8.

[38] Paddick I (2000). A simple scoring ratio to index the conformity of radiosurgical treatment plans Technical note. J Neurosurg.

[39] Lewis D, Chan MF (2015). Correcting lateral response artifacts from flatbed scanners for radiochromic film dosimetry. Med Phys.

[40] Micke A, Lewis DF, Yu X (2011). Multichannel film dosimetry with nonuniformity correction. Med Phys.

[41] Lewis D, Micke A, Yu X, Chan MF (2012). An efficient protocol for radiochromic film dosimetry combining calibration and measurement in a single scan. Medical.

[42] Low DA, Harms WB, Mutic S, Purdy JA (1998). A technique for the quantitative evaluation of dose distributions. Med Phys.

[43] Hansen CR, Crijns W, Hussein M, Rossi L, Gallego P, Verbakel W, et al. Radiotherapy Treatment plannINg study Guidelines (RATING): a framework for setting up and reporting on scientific treatment planning studies. Radiother Oncol 2020;153:67–78. 10.1016/j.radonc.2020.09.033 32976873

[44] Dirix P, Nuyts S, Vander Poorten V, Delaere P, Van den Bogaert W (2008). The influence of xerostomia after radiotherapy on quality of life. Support Care Cancer.

[45] Gayen S, Kombathula SH, Manna S, Varshney S, Pareek P (2020). Dosimetric comparison of coplanar and non-coplanar volumetric-modulated arc therapy in head and neck cancer treated with radiotherapy. Radiat Oncol J.

[46] Mayo C, Martel MK, Marks LB, Flickinger J, Nam J, Kirkpatrick J (2010). Radiation dose-volume effects of optic nerves and chiasm. Int J Radiat Oncol Biol Phys.

[47] Wegner RE, Abel S, Bergin JB, Colonias A (2020). Intensity-modulated radiation therapy in early stage squamous cell carcinoma of the larynx: treatment trends and outcomes. Radiat Oncol J.

[48] Williamson AJ, Bondjie S. Glottic cancer. StatPearls. Treasure Island (FL): StatPearls Publishing. 2021.

[49] Kinshuck AJ, Shenoy A, Jones TM (2017). Voice outcomes for early laryngeal cancer. Curr Opin Otolaryngol Head Neck Surg.

[50] Hussein M, Heijmen BJM, Verellen D, Nisbet A. Automation in intensity modulated radiotherapy treatment planning—a review of recent innovations. Br J Radiol. 2018;91. 10.1259/bjr.20180270 PMC6319857 30074813

[51] Langhans M, Unkelbach J, Bortfeld T (2018). Craft D.

[52] Papp D, Bortfeld T, Unkelbach J. A modular approach to intensity-modulated arc therapy optimization with noncoplanar trajectories. Phys Med Biol 2015;60:5179–98. 10.1088/0031-9155/60/13/5179 26083759

[53] Mullins J, Renaud M-A, Serban M, Seuntjens J (2020). Simultaneous trajectory generation and volumetric modulated arc therapy optimization. Med Phys.

[54] Dong P, Liu H, Xing L. Monte Carlo tree search-based non-coplanar trajectory design for station parameter optimized radiation therapy (SPORT). Phys Med Biol 2018;63. 10.1088/1361-6560/aaca17 29863493

[55] Manser P, Frauchiger D, Frei D, Volken W, Terribilini D, Fix MK (2019). Dose calculation of dynamic trajectory radiotherapy using Monte Carlo. Z Med Phys.

[56] Mueller S, Manser P, Volken W, Frei D, Kueng R, Herrmann E (2018). Part 2: dynamic mixed beam radiotherapy (DYMBER): photon dynamic trajectories combined with modulated electron beams. Med Phys.

