# Supplementary material for: Organ-at-risk sparing with dynamic trajectory radiotherapy for head and neck cancer: comparison with volumetric arc therapy on a publicly available library of cases
Source: Radiat Oncol. 2022 Jul 15;17:122. doi: 10.1186/s13014-022-02092-5 (PMC9284789; doi:10.1186/s13014-022-02092-5)
Supplement: Supplementary file 1 — Additional file 1: A.I. Manual planning rules; Table A.I Optimization objectives for target coverage and conformity; Table A.II. Clinical goals and starting optimization objectives for OARs. A.II. Film measurement protocol; A.III DVH endpoints; Table A.III DVH endpoints for HN1-6; Table A.IV DVH endpoints for HN7. [file 13014_2022_2092_MOESM1_ESM.pdf]

Organ-at-risk sparing with dynamic trajectory radiotherapy for head and neck cancer: comparison with volumetric arc therapy on a publicly available library of cases

Supplementary material

A.I. Manual planning rules

These rules apply to the use of the Eclipse photon optimizer (PO) (Varian Medical Systems)[1]. The goal is to minimize planner bias while enabling to exploit the OAR-sparing potential of each technique by determining objectives that are challenging but nearly achievable.

Before plan optimization

Before plan optimization, the user should choose the number of VMAT arcs or DTRT paths and their associated collimator angle or dynamic collimator paths. The user should decide if parameters such as jaw tracking, avoidance sectors or avoidance structure are to be used. Setting such as convergence mode and aperture shape controller should be clearly selected. In the present study, convergence mode on and aperture shape controller was set to “moderate”. The dose grid was set to 2.5 mm and automatic intermediate dose calculation was used at the multi-resolution (MR) level 4.

Mandatory clinical goals and priorities should be agreed upon prior to optimizations.

Starting objectives

The objectives in table A.I are set before the beginning of the optimization, and it is not allowed to modify them during interactive optimization.

Table A.I: Optimization objectives for target coverage and conformity.

Organ	Objective type	Parameters	Priority
	Normal Tissue Objective (NTO)	Manual<br>- Distance from target border = 0.25 cm<br>- Start dose = 99%<br>- End dose = 30%<br>- Fall-off = 0.25%	100
CTV	Lower	$D_{100\%}$ to $V_{100\%}$	200
PTV	Lower	$D_{100\%}$ to $V_{100\%}$	200
PTV	Lower	$D_{100\%}$ to $V_{95\%}$	200
PTV	Upper	$D_{105\%}$ to $V_{0\%}$	200
Normal Tissue (Body-PTV)	Upper	$D_{100\%}$ to $V_{0\%}$	200

Optimization objectives for the OARs are also set before the optimization based on the clinical goals (Table A.II) but with a priority 0. For the phase 2 and 3 plans, the dose is reduced proportionately to the ratio of prescription dose (e.g. 32% for phase 2 delivering 16 Gy). The objectives in Table A.II are presented in order of priority. Only the spinal cord, brain stem and their respective PRVs have higher priority than target coverage. For all other OARs, if the clinical goals cannot be met, the dose should be reduced as much as possible. The PRV margins were 5 and 3 mm isotropically for spinal cord and brain stem respectively.

Carotid arteries are only considered if outside the target volume with an isotropic PRV margin of 5 mm.

Table A.II: Clinical goals and starting optimization objectives for OARs. Only the dose can be modified manually during the interactive optimization phase.

Organ	Clinical goal	Opt. objective type	Volume [%]	Dose [Gy]
Spinal cord	$D_{0.03\,cc} \leq 45$ Gy [1]	Upper	0	35
PRV spinal cord	$D_{0.03\,cc} \leq 48$ Gy[1]	Upper	0	38
Brainstem	$D_{0.03\,cc} \leq 54$ Gy[1]	Upper	0	43
PRV brainstem	$D_{0.03\,cc} \leq 54$ Gy[1]	Upper	0	43
Hippocampus (R/L)	$V_{7.3\,Gy} \leq 40\%$ [2]	Upper	35	7
Parotid gland (R/L)	$D_{mean} \leq 26$ Gy [3]	Mean		25 /20
Pharynx	$D_{mean} \leq 45$ Gy	Mean		45
	$D_{50\,Gy} \leq 33\%$	Upper	33	50
Larynx GSL	$D_{mean} \leq 35$ Gy	Mean		35
Cochlea (R/L)	$D_{max} \leq 45$ Gy	Upper	0	35
Upper Oesophagus	$D_{mean} \leq 40$ Gy	Mean		40
Lips	$D_{mean} \leq 20$ Gy	Mean		20
	$D_{max} \leq 30$ Gy	Upper	0	30
Oral cavity - PTV	$D_{mean} \leq 40$ Gy[4]	Mean		40
Mandible	$D_{2\%} \leq 70$ Gy	Upper	0	50
Brachial Plexus	$D_{0.03\,cc} \leq 66$ Gy	Upper	0	50
Brain	$D_{0.03\,cc} \leq 65$ Gy	Upper	0	50
		Upper	0.1	40
Eye (R/L)	$D_{0.03\,cc} \leq 45$ Gy	Upper	0	35
	$D_{mean} \leq 35$ Gy	Mean		35
Lachrymal gland (R/L)	$D_{mean} \leq 30$ Gy	Mean		30
Submandibular gland (R/L)	$D_{mean} \leq 35$ Gy	Mean		35
Lens (/R/L)	$D_{0.03\,cc} \leq 6$ Gy	Upper	0	4
Optic chiasm	$D_{0.03\,cc} \leq 54$ Gy	Upper	0	43
Optic nerve (R/L)	$D_{0.03\,cc} \leq 54$ Gy	Upper	0	43
Inner ear (R/L)	$D_{mean} \leq 45$ Gy	Mean		22
Thyroid gland	$V_{50\,Gy} \leq 60\%$	Upper	60	50
		Mean		35
PRV carotid (R/L)	$D_{50\%} \leq 5$ Gy	Upper	47	5
	$D_{30\%} \leq 10$ Gy	Upper	27	10
	$D_{10\%} \leq 15$ Gy	Upper	7	15

[1]: higher priority than target coverage
[2]: introduced for this study
[3]: 20 Gy if only one gland can be spared. Keep below 30 Gy for at least one gland.
[4]: Optimization is performed on oral cavity but plan evaluation is performed on oral cavity excluding PTV

Interactive optimization

Optimization is started in Eclipse with only the objectives of table A.I with non-zero priority. No interaction is permitted until the PO reaches MR level 1, step 3/5. The user then pauses the optimization and sets a priority of 80 to all OAR objectives. Exceptions are made for OARs that have a minimum dose objective (Upper objective to 0% volume) and overlap with the PTV or for OARs with a mean dose objective having a major overlap with the PTV. In this case, the dose value is set to the prescription dose and the priority to 50.

The user resumes the optimization and is allowed only to change the dose value of the OAR objectives. The goal of these interactions is to fulfil the clinical goals and to reduce the dose to the OARs as much as possible, i.e. the objectives must contribute to the objective function

value. After the intermediate dose calculation at MR level 4, the user once again pauses the optimization and can make final adjustments to the dose values of the OAR objectives. The user resumes the optimization again until it completes. Final dose calculation is performed and the user evaluates whether the clinical goals are fulfilled.

Tweaking the objective and re-optimization without manual interaction was allowed if clinical goals were nearly reached. All plans were re-optimized without any manual interaction and the plans were normalized.

A.II. Film measurement protocol

Film measurements were conducted in one end-to-end test on the Alderson phantom. Two Gafchromic EBT3 films (Ashland Advanced Materials, Bridgewater, NJ) were laser-cut to fit between the Alderson slabs closest to target isocenter. The films were scanned 21 hours after irradiation on an Epson XL 10000 flatbed scanner. The scanned films were corrected for lateral response artefact using a one-dimensional linear correction function [2]. Triple channel calibration was used to convert colour values to absolute dose [3]. Dose rescaling was applied according to the one-scan protocol using two addition film strips [4]. The resulting dose to the red channel was used for comparison with the corresponding 2D calculated dose plane in Eclipse using gamma evaluation with a 2% (global)/2mm criterion and a 10% dose threshold of the maximum dose [5].

A.III / A.IV DVH endpoints

Table A.III reports clinical goals and DVH endpoints for HN1-6 treated with sequential boost techniques. Table A.IV reports clinical goals and DVH endpoints for HN7, single vocal cord irradiation treated according to the VoiceS protocol (NCT04057209, available upon request). Endpoints above tolerance are shown in red. As per institutional guidelines, these clinical goals are not mandatory but the dose should be reduced as much possible and the following variations are acceptable:

- Parotid glands: mean dose below 30 Gy for at least one gland,
- Pharynx: mean dose below 55 Gy,
- Oral cavity (excluding PTV): mean dose below 50 Gy,
- Brachial plexus and brain: $D_{0.03cc}$ below 70 Gy
- Mandible: $D_{2\%}$ below 75 Gy

Table A.III DVH endpoints for HN1-6

OAR	Endpoint / goal [Gy/%]	Dose [Gy] or volume [%] - VMAT / DTRT					
		HN1	HN2	HN3	HN4	HN5	HN6
**Target**							
PTV70 $D_{95\%}$	70.0 Gy[1]	71.0 / 71.0	70.5 / 70.7	70.3 / 70.1	NA	NA	70.2 / 70.3
PTV70 $D_{5\%}$	$\leq$ 74.9 Gy	73.9 / 74.0	73.3 / 73.5	73.5 / 73.7	NA	NA	73.1 / 72.9
PTV66 $D_{95\%}$	$\geq$ 66.0 Gy[2]	NA	69.5 / 69.4	NA	66.5 / 66.4	66.0 / 65.9	NA
PTV66 $D_{98\%}$	$\geq$ 62.7 Gy	NA	68.6 / 68.4	NA	66.2 / 66.1	65.2 / 65.1	NA
PTV66 $D_{5\%}$	$\leq$ 70.6 Gy	NA	NA	NA	68.6 / 68.6	69.1 / 69.2	NA
PTV50 $D_{95\%}$	$\geq$ 50.0 Gy	51.5 / 52.8	51.1 / 50.4	50.5 / 51.1	51.6 / 52.4	62.4 / 60.5	63.4 / 63.6
PTV50 $D_{98\%}$	$\geq$ 47.5 Gy	50.9 / 51.9	50.4 / 51.3	50.0 / 50.6	51.0 / 51.7	60.7 / 58.1	60.0 / 60.9
**Salivary and swallowing**							
Contr. parotid	$D_{mean} \leq 26$	26.3 / 26.8	16.0 / 13.1	10.8 / 8.1	7.5 /1.1	4.6 / 2.2	0.3 / 0.3
Ips. parotid	$D_{mean} \leq 26$	28.1 / 28.5	23.4 / 23.8	23.3 / 2.2	Resected	7.4 / 4.0	0.3 / 0.3
Contr. submand.	$D_{mean} \leq 35$	20.4 / 19.5	21.8 / 16.9	16.0 / 13.4	2.6 / 2.2	0.5 / 3.2	1.4 / 1.5
Ips. submand.	$D_{mean} \leq 35$	NA	Resected	31.4 / 31.4	7.9 / 9.3	0.5 / 4.4	1.2 / 1.2
Pharynx	$D_{mean} \leq 45$	51.3 / 49.4	51.0 / 49.8	41.2 / 41.1	16.3 / 12.8	1.4 / 6.7	9.2 / 10.6
Oral cavity - PTV	$D_{mean} \leq 40$	29.9 / 25.6	40.1 / 36.0	34.4 / 33.0	11.6 / 9.1	2.0 / 11.2	0.4 / 0.4
Larynx GSL	$D_{mean} \leq 35$	31.6 / 25.1	34.1 / 34.2	27.7 / 29.1	0.7 / 3.1	0.3 / 2.7	NA
**Auditory and optical**							
Contr. cochlea	$D_{0.03\,cc} \leq 45$	6.5 / 11.8	3.5 / 8.6	1.8 / 4.3	3.9 / 2.3	12.0 / 5.9	<= 0.1
Ips. cochlea	$D_{0.03\,cc} \leq 45$	11.2 / 18.6	4.3 / 13.2	2.9 / 17.4	16.3 / 15.5	9.7 / 9.7	<= 0.1
Contr. inner ear	$D_{mean} \leq 45$	5.5 / 10.2	3.0 / 8.4	1.6 / 3.9	3.1 / 2.0	9 / 4.2	<= 0.1
Ips. inner ear	$D_{mean} \leq 45$	7.7 / 16.3	3.6 / 12.8	2.6 / 14.4	14.8 / 14.4	9.6 / 8.6	<= 0.1
Contr. eye	$D_{0.03\,cc} \leq 45$	7.8 / 8.4	4.1 / 11.4	2.2 / 5.0	6.6 / 3.7	26.9 / 26.3	<= 0.1
Contr. eye	$D_{mean} \leq 35$	3.2 / 6.1	1.8 / 6.8	1.2 / 3.5	2.3 / 2.1	12.9 / 9.6	<= 0.1
Ips. eye	$D_{0.03\,cc} \leq 45$	7.0 / 7.1	4.2 / 7.5	2.6 / 6.7	10.7 / 10.5	41.0 / 41.7	<= 0.1
Ips. eye	$D_{mean} \leq 35$	2.9 / 4.7	1.8 / 4.3	1.3 / 4.3	3.1 / 5.8	14.5 / 12.8	<= 0.1
Contr. lachrymal	$D_{mean} \leq 30$	1.9 / 5.8	1.2 / 6.9	0.8 / 4.6	1.2 / 1.1	11.4 / 5.6	<= 0.1
Ips. lachrymal	$D_{mean} \leq 30$	1.9 / 4.0	1.3 / 3.3	0.9 / 3.5	1.5 / 7.7	11.6 / 7.7	<= 0.1
Contr. lens	$D_{0.03\,cc} \leq 6$	3.4 / 5.4	1.8 /5.5	1.2 / 2.9	2.2 / 2.6	7.2 / 6.4	<= 0.1
Ips. lens	$D_{0.03\,cc} \leq 6$	3.0 / 4.7	1.8 / 3.6	1.3 / 3.4	2.7 / 4.4	8.2 / 7.2	<= 0.1
Optic chiasm	$D_{0.03\,cc} \leq 54$	1.9 / 9.9	1.4 / 8.7	1.1 / 9.8	1.2 / 3.7	10.7 / 11.4	<= 0.1
Contr. optic nerve	$D_{0.03\,cc} \leq 54$	2.1 / 10.9	1.6 /10.4	1.3 / 6.0	1.3 / 3.1	31.4 / 20.2	<= 0.1
Ips. optic nerve	$D_{0.03\,cc} \leq 54$	2.5 / 11.0	1.8 / 13.1	1.3 / 10.2	1.8 / 13.8	54.8 / 53.3	<= 0.1
**Nervous and circulatory**							
Spinal cord	$D_{0.03\,cc} \leq 45$	36.5 / 31.3	32.0 / 30.5	31.6 / 31.8	25.4 / 14.3	17.1 / 15.7	22.9 / 23.4
PRV spinal cord	$D_{0.03\,cc} \leq 48$	41.1 / 36.9	34 / 35.1	35.4 / 36.8	28 / 18.1	18.3 / 16.8	27.6 / 26.7
Brainstem	$D_{0.03\,cc} \leq 54$	19.8 / 22.1	14.7 / 22.5	16.9 / 19.9	19.0 / 11.3	25.8 / 22.	0.2 / 0.2
PRV brainstem	$D_{0.03\,cc} \leq 54$	22.0 / 26.2	16.4 / 25.5	18.9 / 22.7	21.1 / 13.2	26.9 / 23.7	0.2 / 0.2
Brachial Plexus	$D_{0.03\,cc} \leq 66$	68.3 / 68.7	56.6 / 55.8	52.6 / 53.6	1.4 / 14.1	0.3 / 3.0	35.7 / 39.2
Brain	$D_{0.03\,cc} \leq 65$	32.6 / 37.5	24.7 / 36.0	20.4 / 32.7	49.8 / 47.9	69.7 / 69.1	0.2 / 0.2
Contr. carotid PRV	$D_{50\%} \leq 5$	NA	NA	5.1 / 5.6	0.9 / 1.0	0.3 / 1.3	0.7 / 0.8
Contr. carotid PRV	$D_{30\%} \leq 10$	NA	NA	7.5 / 6.8	5.0 / 2.1	0.8 / 1.7	1.4 / 2.0
Contr. carotid PRV	$D_{10\%} \leq 15$	NA	NA	13.8 / 12.1	11.7 / 3.8	14.7 / 11.1	15.0 /13.5
Ips. carotid PRV	$D_{50\%} \leq 5$	NA	NA	NA	NA	0.4 / 6.2	
Ips. carotid PRV	$D_{30\%} \leq 10$	NA	NA	NA	NA	0.9 / 3.8	
Ips. carotid PRV	$D_{10\%} \leq 15$	NA	NA	NA	NA	14.7 / 14.2	
**Other structures**							
Contr. hippo.	$V_{7.3\,Gy} \leq 40$	0 / 8.8	0 /3.3	0 / 0	0 / 0	20.5 / 3.4	0 / 0
Ips. hippo.	$V_{7.3\,Gy} \leq 40$	0 / 3.4	0 / 28.5	0 / 22.2	0 / 6.9	38.0 / 30.5	0 / 0
Upper oesophagus	$D_{mean} \leq 40$	12.0 / 14.4	14.3 / 13.5	8.6 / 8.7	0.3 / 1.3	0.1 / 2.0	18.0 / 15.4
Mandible	$D_{2\%} \leq 70$	72.2 / 72.6	72.4 / 72.9	61.4 / 59.3	66.7 / 66.0	31.4 / 19.7	0.6 / 0.6
Lips	$D_{mean} \leq 20$	19.8 / 16.3	24.6 / 21.9	17.8 / 14.9	8.0 / 6.3	1.1 / 6.4	0.2 / 0.2
Lips	$D_{max} \leq 30$	39.3 / 35.2	71.5 / 74.5	30.5 / 28.2	17.6 / 23.0	2.6 / 17.0	0.4 / 0.4
Thyroid	$V_{50\,Gy} \leq 60$	15.3 / 20.1	18.5 / 26.9	3.1 / 5.1	0 / 0	0 / 0	14.7 / 13.8

[1]: within +/- 2%, [2]: within +/-2% for HN 4-5

Abbreviations: contr: contralateral, ips.: ipsilateral, submand.: submandibular gland. PRV: planning at risk volume.

Table A.IV: Dosimetric endpoints for HN7 (single vocal cord irradiation)

PTV objective	Deviation acceptable	Dose [Gy] VMAT / DTRT
$D_{99\%}$ = 58.08 Gy	≥ 55.176 Gy (95%)	56.34 / 56.34
$D_{1\%}$ ≤ 60.98 Gy	≤ 62.15 Gy	61.52 / 61.34
$D_{0.03cc}$ ≤ 62.15 Gy	≤63.89 Gy	61.97 / 61.61
OAR	Endpoint/ objective	
Normal Tissue	$D_{0.03cc}$ ≤ 60.98 Gy (62.15 Gy acceptable)	54.96 / 55.15
Spinal cord*	D_{max} ≤ 30 Gy	16.2 / 14.9
PRV spinal cord*	D_{max} ≤ 35 Gy	18.4 / 17.8
Contr. Vocal cord	D_{mean} ≤ 50 Gy	40.7 / 36.4
Larynx	D_{mean} ≤ 40 Gy	29.6 / 31.1
Contr. Arytenoid	D_{mean} ≤ 40 Gy	21.1 / 13.3
Mid constrictor	D_{mean} ≤ 40 Gy	2.2 / 4.0
Inf constrictor	D_{mean} ≤ 40 Gy	20.5 / 13.8
Supra-larynx	D_{mean} ≤ 20 Gy	17.8 / 20.0
Cricophayngeal muscle	D_{mean} ≤ 40 Gy	23.4 / 22.7
Brachial plexus*	D_{max} ≤ 45 Gy	22.6 / 16.9
Ipsi. Carotid	D_{max} ≤ 30 Gy	26.4 / 23 .2
Contr. carotid	D_{max} ≤ 15 Gy	12.9 / 11.7
Thyroid gland	D_{mean} ≤ 15 Gy	15.2 / 14.4

* mandatory goal

A.V RATiNG

The RATiNG guidelines for treatment planning studies [6] were followed and the final score was 96%. The score sheet is attached as supplementary material.

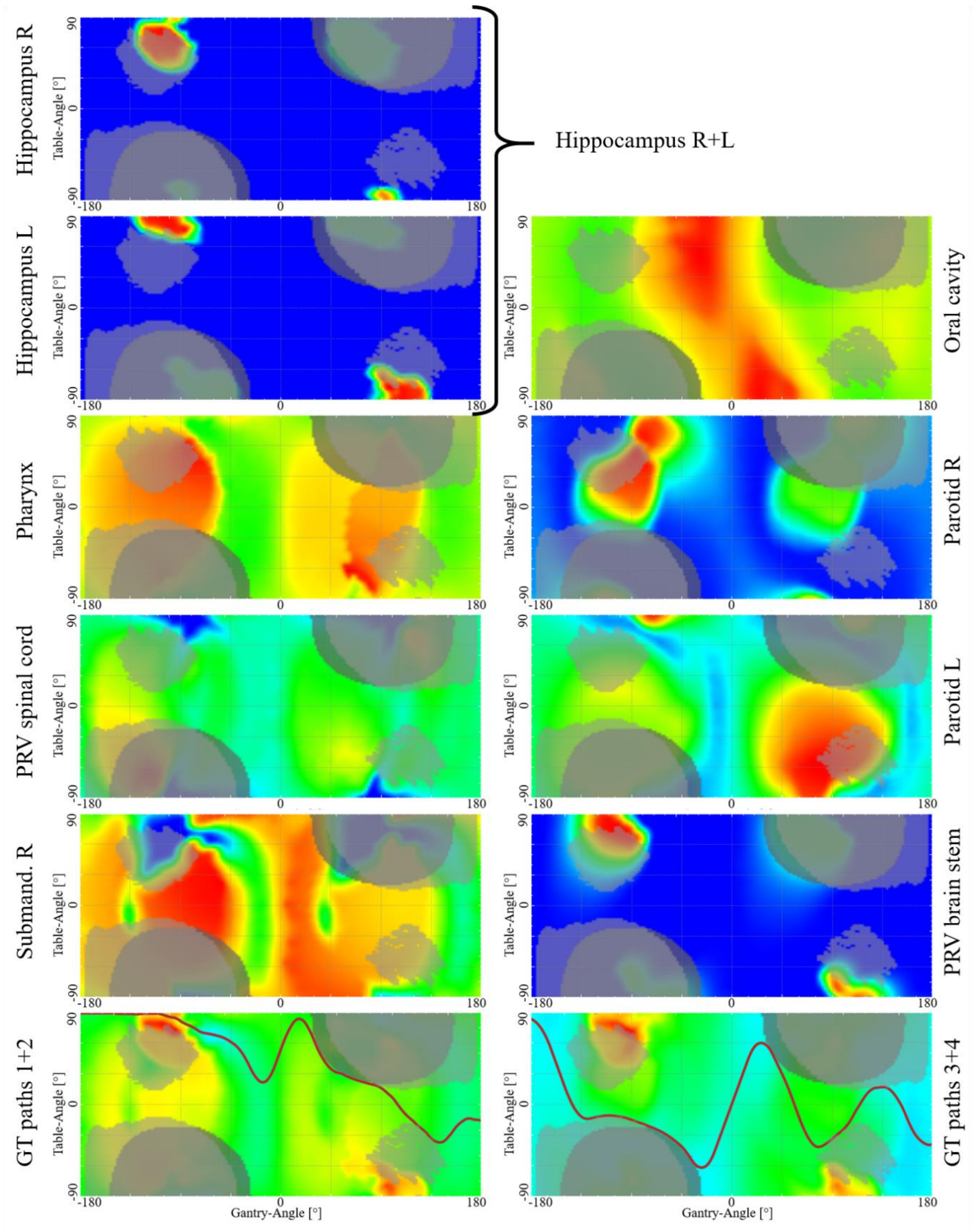

Supplementary Figure 1: Individual OAR GT-maps and map sum for each path for HN2. The hippocampus R+L was included in both maps.

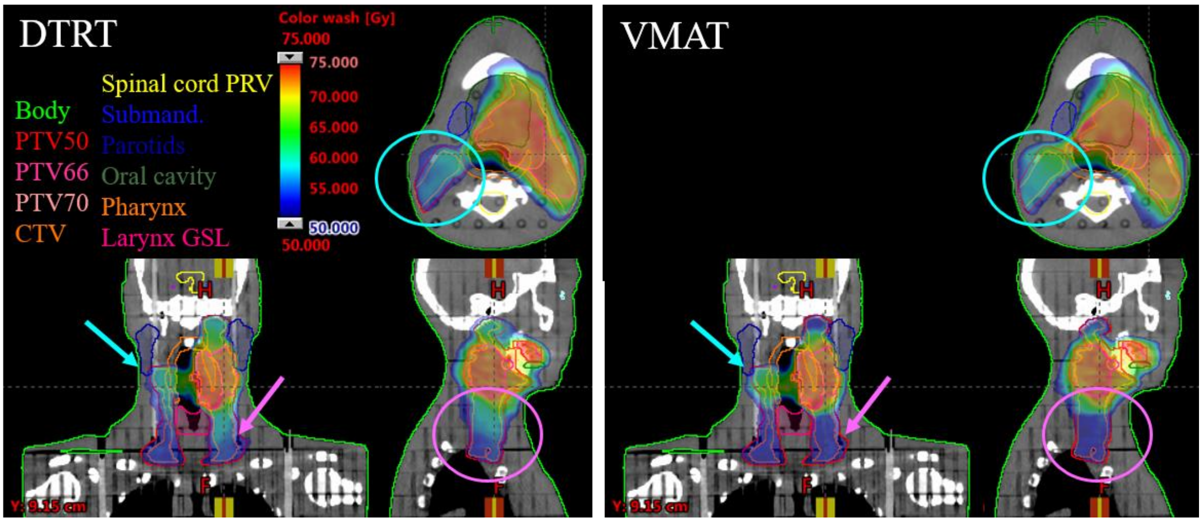

Supplementary Figure 2: Dose distribution for HN2. Where the elective nodal volume (PTV50) extends the high dose volume (PTV66/70) in the axial direction, PTV50 receives more dose with VMAT than DTRT (cyan circle and arrow). On the contrary, where PTV50 extends PTV66/70 inferiorly, PTV50 receives more dose with DTRT than VMAT (magenta circle and arrows).

References

[1] Varian Medical Systems. Eclipse 15.5 Photon and Electron Algorithms Reference Guide 2017.

[2] Lewis D, Chan MF. Correcting lateral response artifacts from flatbed scanners for radiochromic film dosimetry. Med Phys 2015;42:416–29. https://doi.org/10.1118/1.4903758.

[3] Micke A, Lewis DF, Yu X. Multichannel film dosimetry with nonuniformity correction. Med Phys 2011;38:2523–34. https://doi.org/10.1118/1.3576105.

[4] Lewis D, Micke A, Yu X, Chan MF. An efficient protocol for radiochromic film dosimetry combining calibration and measurement in a single scan. Medical 2012;39:6339–50.

[5] Low DA, Harms WB, Mutic S, Purdy JA. A technique for the quantitative evaluation of dose distributions. Med Phys 1998;25:656–61. https://doi.org/10.1118/1.598248.

[6] Hansen CR, Crijns W, Hussein M, Rossi L, Gallego P, Verbakel W, et al. Radiotherapy Treatment plannINg study Guidelines (RATING): A framework for setting up and reporting on scientific treatment planning studies. Radiother Oncol 2020;153:67–78. https://doi.org/10.1016/j.radonc.2020.09.033.
