# Supplementary material for: Organ-at-risk sparing with dynamic trajectory radiotherapy for head and neck cancer: comparison with volumetric arc therapy on a publicly available library of cases
Source: Radiat Oncol. 2022 Jul 15;17:122. doi: 10.1186/s13014-022-02092-5 (PMC9284789; doi:10.1186/s13014-022-02092-5)
Supplement: Supplementary file 2 — Additional file 2: A.V. RATiNG. [file 13014_2022_2092_MOESM2_ESM.pdf]

RATING score sheet

		Points	Applicable/ relevant	Answer yes
**Questions for the Introduction**				
	The study aim formulated by research questions			
1	Does the study have a concise and precise study aim, defined with a restricted number of interconnected questions?	10		☑
	The motivation for the research questions			
2	Has relevant up to date literature been included to support the need for the current study?	5		☑
3	Does the study address an existing knowledge gap?	10		☑
**Questions for Materials and Methods**				
4	Is the global study design adequate for answering the posed research questions?	10		☑
5	Is the global study design described in sufficient detail for others to interpret and reproduce the results?	5		☑
	Patient cohort			
6	Are the inclusion and exclusion criteria of the patient cohort described?	1	☑	☐
7	Is the clinical patient information of the cohort presented, including disease type, site(s) and clinical staging?	1	☑	☐
8	Is the included number of patients stated, explained and justified?	1	☑	☑
9	Has there been consideration of the need for ethical and/or legal approval for the study and if needed, is there a statement about this?	5		☑
	Imaging procedures			
10	Have the scanning parameters been reported in sufficient detail (image modalities, equipment model, slice thickness, voxel size, patient position (e.g. head first, supine, etc.) etc.)?	1	☑	☑
11	Has the applied immobilisation equipment been described, (e.g. vendor and type, standard settings, etc.) where relevant?	1	☑	☑
	Treatment machine and settings			
12	Have the treatment machine and relevant parameters been described with sufficient detail (model, beam energy, MLC, etc.)?	1	☑	☑
13	Have the monitor unit reference conditions been defined, where relevant?	1	☐	☐
	Definition of targets and OARs			
14	Has GTV definition been described in sufficient detail, with references if possible?	1	☐	☐
15	Has CTV definition been described in sufficient detail, with references if possible?	1	☑	☑
16	Has the establishment of PTVs (or alternatively robustness settings) been described in sufficient detail?	1	☑	☑
17	Have PTV sizes in the patient cohort been described?	1	☑	☑
18	Have OAR definitions been described in sufficient detail, with references if possible?	1	☑	☑
19	Have PRV margins been described in sufficient detail, with references if available?	1	☑	☑
	Treatment planning system and dose calculation			

20	Have all applied dose calculation algorithms been described in sufficient detail?	1	☑	☑
21	For any commercial software used, have the manufacturer, algorithms and specific versions been stated?	1	☑	☑
22	Have all relevant user parameters and settings in the TPS been reported, e.g. beams, dose grid, control point spacing?	1	☑	☑
23	Have all volumes been evaluated with the same software/methodology?	1	☑	☑
	Planning aims and optimisation			
24	Are clear planning aims defined, including imposed hard constraints and planning objectives (with or without soft constraints)?	5		☑
25	Has the ranking of planning objectives (priorities) been described?	5		☑
26	Is the dose prescription clearly defined?	10		☑
27	Is there a narrative description of the applied optimisation process, including the handling of all objectives with their ranking?	5		☑
28	If manual intervention during or after optimisation is allowed, has this been described?	1	☑	☑
	Bias mitigation			
29	Have enough study details been provided such that bias issues could be noted?	5		☑
30	Has bias been sufficiently mitigated to reliably answer the posed research question?	10		☑
	Plan acceptability – minor and major protocol deviations			
31	Was the procedure for assessment of plan acceptability well-described?	1	☑	☑
32	Was the procedure for assessment of minor and major protocol deviations well described?	1	☑	☑
	Plan (re-)normalisation for plan comparisons			
33	Has plan (re-)normalisation been described sufficiently?	1	☑	☑
	Dose-volume parameters for plan evaluation and comparison			
34	Have sufficiently comprehensive dose-volume parameters been used for plan evaluations and comparisons?	5		☑
	Population-mean DVHs			
35	Has the algorithm for creating population-mean/median DVHs been reported?	1	☐	☐
36	Have the definitions of confidence intervals been included?	1	☐	☐
	Plan evaluations by clinicians			
37	Have clinicians scored plans to assess quality?	1	☐	☐
38	Were plan comparisons by clinicians blinded?	1	☐	☐
	Predicted tumour control probability and normal tissue complication probabilities for plan evaluation and comparison			
39	Have any applied TCP models been described and referenced?	1	☐	☐
40	Have any applied NTCP models been described and referenced?	1	☐	☐
	Plan deliverability and complexity			
41	Have methods used to assess plan deliverability and complexity been described in sufficient detail?	1	☑	☑
	Composite plan quality metrics			
42	Is there a sufficient basis (e.g. in the literature) for any selected composite plan quality metrics?	1	☐	☐

43	Is there an adequate description of the calculation of the composite plan quality metrics?	1	☐	☐
	Planning and delivery times			
44	Has measurement of planning times been described in sufficient detail?	1	☑	☑
45	Has the establishment of delivery times been described in sufficient detail?	1	☑	☑
	Statistical analysis			
46	Have proper statistical methods been used and described in sufficient detail?	5		☐
47	In case of multiple testing for research questions, has this been handled appropriately?	1	☐	☐
	**Questions for Results**			
48	Does the provided data contribute to (at least partly) answering all aspects of the research questions, e.g. plan acceptability, dosimetric quality, deliverability and planning and delivery times?	10		☑
	Dose distribution reporting			
49	Are complete summaries of the dose distributions in the patient cohort provided (low doses, high doses, OARs, PTV, patient, etc.)?	5		☑
50	Are tables and figures optimised to clearly present the results obtained?	1	☑	☑
51	Have the answers to the research questions been illustrated for an example patient by providing dose distributions, DVHs, etc.?	1	☑	☑
	Plan acceptability reporting – minor and major protocol deviations			
52	In case of treatment technique or planning technique comparisons, was plan acceptability reported separately for each technique?	1	☑	☑
53	Has plan acceptability been reported in sufficient detail: how many plans were acceptable, how many were not and for what reasons (e.g. violation of hard constraints, violation of soft constraints, other reasons)?	1	☑	☑
54	Was there adequate reporting of minor and major protocol deviations?	1	☑	☑
	Deliverability and complexity reporting			
55	Has the deliverability of the plans been adequately reported?	1	☑	☑
56	Have plan deliverability and complexity been investigated in sufficient detail in relation to the posed research questions?	1	☑	☑
	Planning and delivery times reporting			
57	Have planning and delivery times been adequately evaluated and reported?	1	☑	☑
	Patient-specific analyses reporting			
58	Is there sufficient description of inter-patient variations in the results presented?	1	☑	☑
59	Have outlier patients been reported and has any exclusion from population analyses been sufficiently motivated and explained?	1	☑	☑
	Statistical reporting			
60	Are the p-values reported appropriately?	1	☐	☐
61	Are there confidence intervals for the appropriate parameters?	1	☐	☐
	**Questions for discussions**			

62	Is there an overall interpretation of the data presented in the Results section as to how the posed research questions are answered?	10		☑
	Comparison with literature			
63	Has the study been sufficiently discussed in the context of existing literature?	5		☑
	Clinical and statistical significance			
64	Does the discussion focus on statistically significant results?	1	☑	☐
65	Is the potential clinical significance of the results clearly discussed (assuming practical application would be feasible)?	5		☑
	Clinical applicability of the study			
66	Is future the clinical applicability sufficiently discussed?	1	☑	☑
	Study limitations			
67	Has the impact of the study limitations on the provided answers to the research questions been sufficiently discussed?	10		☑
	Future work			
68	Has the potential future work arising from the study been discussed?	1	☑	☑
**Questions for conclusions**				
69	Do the presented conclusions represent answers to the posed research questions?	5		☑
70	Are the conclusions fully supported by the results?	5		☑
71	Are the conclusions a fair summary of all results?	5		☑
**Questions for supplementary**				
	Supplementary materials			
72	Is the information presented in the supplementary material of sufficient relevance?	1	☑	☑
73	Is the presentation of the included information of sufficient quality, including readability?	1	☑	☑
74	Has sufficient underlying data been made available or a willingness to share data been indicated, within local data sharing restrictions?	5		☑
**RATING remarks**				
75	Is the RATING score added to the manuscript?	5		☑
76	Is the accompanying question table added to the cover letter or the supplementary material?	1	☑	☑

RATING score		96%	
**RATING fraction**	195	of	203
